# Distinct seasonal dynamics of responses to elevated CO_2_ in two understorey grass species differing in shade‐tolerance

**DOI:** 10.1002/ece3.5738

**Published:** 2019-11-29

**Authors:** Petr Holub, Karel Klem, Sune Linder, Otmar Urban

**Affiliations:** ^1^ Global Change Research Institute of the Czech Academy of Sciences Brno Czech Republic; ^2^ Southern Swedish Forest Research Centre Swedish University of Agricultural Sciences Alnarp Sweden

**Keywords:** *Calamagrostis arundinacea*, ecological niche, glass domes, light environment, *Luzula sylvatica*, manipulation experiment, seasonal dynamics

## Abstract

1. Understorey plant communities are crucial to maintain species diversity and ecosystem processes including nutrient cycling and regeneration of overstorey trees. Most studies exploring effects of elevated CO_2_ concentration ([CO_2_]) in forests have, however, been done on overstorey trees, while understorey communities received only limited attention.
2. The hypothesis that understorey grass species differ in shade‐tolerance and development dynamics, and temporally exploit different niches under elevated [CO_2_], was tested during the fourth year of [CO_2_] treatment. We assumed stimulated carbon gain by elevated [CO_2_] even at low light conditions in strongly shade‐tolerant *Luzula sylvatica*, while its stimulation under elevated [CO_2_] in less shade‐tolerant *Calamagrostis arundinacea* was expected only in early spring when the tree canopy is not fully developed.
3. We found evidence supporting this hypothesis. While elevated [CO_2_] stimulated photosynthesis in *L. sylvatica* mainly in the peak of the growing season (by 55%–57% in July and August), even at low light intensities (50 µmol m^−2^ s^−1^), stimulatory effect of [CO_2_] in *C. arundinacea* was found mainly under high light intensities (200 µmol m^−2^ s^−1^) at the beginning of the growing season (increase by 171% in May) and gradually declined during the season. Elevated [CO_2_] also substantially stimulated leaf mass area and root‐to‐shoot ratio in *L. sylvatica*, while only insignificant increases were observed in *C. arundinacea*.
4. Our physiological and morphological analyses indicate that understorey species, differing in shade‐tolerance, under elevated [CO_2_] exploit distinct niches in light environment given by the dynamics of the tree canopy.

## Graphical abstract

Our data support the hypothesis that elevated [CO_2_] stimulates carbon uptake and growth of strong shade‐tolerant understorey grass species, while only short‐term increases were found in sun‐demanding species during the spring months. The hypothesis that understorey species, differing in shade‐tolerance, exploit under elevated [CO_2_] distinct light niches was supported. Presented research contributes to our understanding of a complex response of forest ecosystems and changes in biodiversity of understorey vegetation in temperate zone under the conditions of increasing atmospheric CO_2_ concentration.

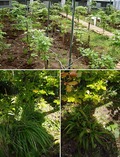

## 1 INTRODUCTION

In order to predict the responses of natural plant communities to future increases in atmospheric CO_2_ concentration ([CO_2_]), it is necessary to understand the different responses of the species and ecosystems to elevated [CO_2_] and the ability of species to use newly established niches. This is particularly important for the understorey species since the light limitation can strongly affect their response to [CO_2_]. Previous studies have shown that elevated [CO_2_] often stimulates growth (e.g., de Graaff, van Groenigen, Six, Hungate, & van Kessel, 2006; Poorter, 1993) and photosynthesis (e.g., Albert et al., 2011), reduces stomatal conductance (Ainsworth & Rogers, 2007), increases water use efficiency (Curtis & Wang, 1998), and increases growth of the root system, particularly root length, and root‐to‐shoot ratio (Anderson et al., 2010; Rogers, Peterson, McCrimmon, & Cure, 1992). Such physiological and anatomical modifications under elevated [CO_2_] may increase water use efficiency in plants and reduce thus the adverse effects of drought stress (Ainsworth & Rogers, 2007; Tschaplinski, Stewart, Hanson, & Norby, 1995; Wang et al., 2018).

Most of the studies exploring effects of elevated [CO_2_] in forest ecosystems have, however, been done on dominant overstorey trees under conditions of high light intensities (e.g., Asshoff, Zotz, & Körner, 2006; Norby et al., 2005; Urban et al., 2014), while understorey communities, naturally exposed to very low daily light integrals—photosynthetic photon flux density integrated over a day (DLI), received so far only limited attention (Belote, Weltzin, & Norby, 2004; Dukes et al., 2005; Niklaus & Körner, 2004; Würth, Winter, & Körner, 1998). These communities are, however, crucial to maintain species diversity, the stability of the habitat, and other ecosystem processes including regeneration of overstorey trees and nutrient cycling (Gilliam & Roberts, 2003). Understorey vegetation also plays a crucial role in utilizing new niches arising under changing environmental conditions (Gilbert & Lechowicz, 2004).

Daily light integral has significant impacts on a range of leaf/plant traits related to anatomical structure, chemical composition, physiological responses, and growth (Augspurger, Cheeseman, & Salk, 2005; Hättenschwiler, 2001; Lichtenthaler, Ač, Marek, Kalina, & Urban, 2007; Rajsnerová et al., 2015). Meta‐analysis study of 70 leaf traits has shown that these changes are generally larger at low DLIs, while tend to saturate at high DLI (Poorter et al., 2019). Among others, leaf mass per area (LMA) and leaf thickness increase with increasing DLI, that is, the parameters increasing also with increasing [CO_2_]. In contrary, increases in Rubisco carboxylation rate and Rubisco content associated with increasing DLI could be substantially reduced under long‐term exposure to elevated [CO_2_] (Ceulemans & Mousseau, 1994; Leakey et al., 2009; Norby, Warren, Iversen, Medlyn, & McMurtrie, 2010; Urban, 2003; Way, Oren, & Kroner, 2015). Such examples suggest a possible interaction between DLI and [CO_2_] ranging from synergistic to antagonistic effects.

Indeed, reports of CO_2_ stimulating effects on photosynthesis and related processes under low light intensities are contradictory. Urban et al. (2014) found reduced carbon gain and light use efficiency in temperate beech trees grown under elevated [CO_2_] during cloudy sky conditions accompanied by low light intensity, low temperature, and high air humidity. In contrary, it has been shown that elevated [CO_2_] stimulates the rate of photosynthetic CO_2_ uptake under the conditions of deep shade and high temperature in the understorey of a tropical rain forest (Würth et al., 1998). Such sensitivity to [CO_2_] is predicted to be caused by reduced photorespiratory carbon loss, increased apparent quantum efficiency, and accordingly reduced the light compensation irradiance of photosynthesis under elevated [CO_2_] (Drake, Gonzalez‐Meler, & Long, 1997; Farquhar, Caemmerer, & Berry, 1980; Hättenschwiler & Körner, 1996, 2000). All these studies, however, suggest that photosynthetic rate is modulated by combined conditions of elevated [CO_2_] and low light intensities and may thus potentially alter the carbon balance of understorey plants as well as species composition.

A meta‐analysis by Kerstiens (2001) revealed a significantly higher increase of biomass under elevated [CO_2_] in shade‐tolerant as compared to shade‐intolerant species. In contrary, DeLucia and Thomas (2000) did not find the correlation between the stimulation of light‐saturated photosynthesis by elevated [CO_2_] and shade‐tolerance ranking of four tree species growing in the understory of a loblolly pine plantation. The different responses of shade‐tolerant and shade‐intolerant species are obvious only at high DLI values which in understorey can be achieved during the summer months or in a not completely closed canopy allowing higher frequency of sunflecks (Naumburg & Ellsworth, 2000). Particularly for deciduous and mixed forests, distinct light niches for understorey vegetation are available (Augspurger et al., 2005; Gilbert & Lechowicz, 2004). The first is represented by early spring with an open canopy before leaf out, which can be exploited by species with fast development, ability to utilize higher light intensities, and to survive under later deep shade. The second niche is exploited by typically shade‐tolerant species, using mainly the higher DLIs during the summer months.

Such inconsistent results of responses of understorey vegetation to elevated [CO_2_] may further rise from differences in soil water availability. For example, Belote et al. (2004) observed stimulatory effect of elevated [CO_2_] on aboveground biomass production of Nepal grass (*Microstegium vimineum*)—an understorey dominant species in a dry, but not in a wet year.

In the present study, we explored responses of growth and photosynthesis to elevated [CO_2_] in two grass species with C3 photosynthetic pathway grown in the understorey of an experimental spruce‐beech stand. The studied grasses, *Calamagrostis arundinacea* (L.) Roth and *Luzula sylvatica* (Huds.) Gaud., represent widespread species of montane forests in Central Europe. Tuft forming *C. arundinacea* is an expansive and sun‐demanding species occurring in the majority of disturbed forests and open deforested areas (Fiala, Tůma, Holub, & Jandák, 2005; Fiala et al., 2001). On the other hand, rhizomatous *L. sylvatica* is a highly shade‐tolerant species, widespread over the temperate zone, and typically occurring in deep forest understories at low DLI (Godefroid, Rucquoij, & Koedam, 2005).

We tested the hypothesis that (a) elevated [CO_2_] stimulates photosynthesis and growth of understorey plant species under natural low light intensities. More specifically, we have assumed that (b) species differing in shade‐tolerance also have a different sensitivity to elevated [CO_2_] due to a different composition and operation of the photosynthetic apparatus. Finally, we expected that (c) the stimulation effects of elevated [CO_2_] are changing throughout the growing season following the changes in DLI and development of forest canopies enabling thus the species differing in shade‐tolerance to use distinct niches in the light environment.

## 2 MATERIALS AND METHODS

### 2.1 Experimental plants and design

At the beginning of the growing season 2007, tillers of *C. arundinacea* and *L. sylvatica* were collected from an open area near the experimental station Bílý Kříž (Czech Republic; 49°33′N 18°32′E, 908 m a.s.l.) and subsequently exposed for four growing seasons to ambient (385 µmol CO_2_/mol; AC) and elevated (700 µmol CO_2_/mol; EC) [CO_2_] using the glass domes at Bílý Kříž (see Figure S1, Šigut et al. (2015) and Urban et al. (2001) for technical description of the experimental facilities). The plants were investigated during the fourth growing season (2010) under the controlled growth [CO_2_] conditions.

Fifteen transplanted plant tufts of both grass species per treatment were planted in the understorey of a 10‐year‐old mixed spruce‐beech stand (*Picea abies* (L.) Karst. and *Fagus sylvatica* (L.)). Seasonal maxima of projected leaf area index, estimated by a LAI‐2000 Plant Canopy Analyser (Li‐Cor) in AC and EC stands, are shown in Table 1. Plants with comparable biomass and developmental stage were transplanted (data not shown). Plants were grown in the native soil. The geological bedrock is formed by Mesozoic Godula sandstone (flysch type) and is overlain by Ferric Podzols. The total soil nitrogen was found to range between 2.7 and 3.5 mg/g irrespective of [CO_2_] treatment. Plants within each dome were split into five blocks (replications). Each block consisted of three plants of *C. arundinacea* and three plants of *L. sylvatica*. Two plants per block were evaluated, and the average from these two measurements was used for statistical analyses.

**Table 1:** Seasonal maxima of projected leaf area index (LAI; m^2^/m^2^) estimated in mixed spruce‐beech experimental stands cultivated under ambient (AC) and elevated (EC) CO_2_ concentration during three consecutive years

	2008	2009	2010
AC stand	1.18 ± 0.25	1.77 ± 0.34	2.16 ± 0.35
EC stand	1.23 ± 0.27	1.87 ± 0.36	2.38 ± 0.41

Note

Mean values ± standard deviations (*n* = 8) are shown.

**Figure 1:**
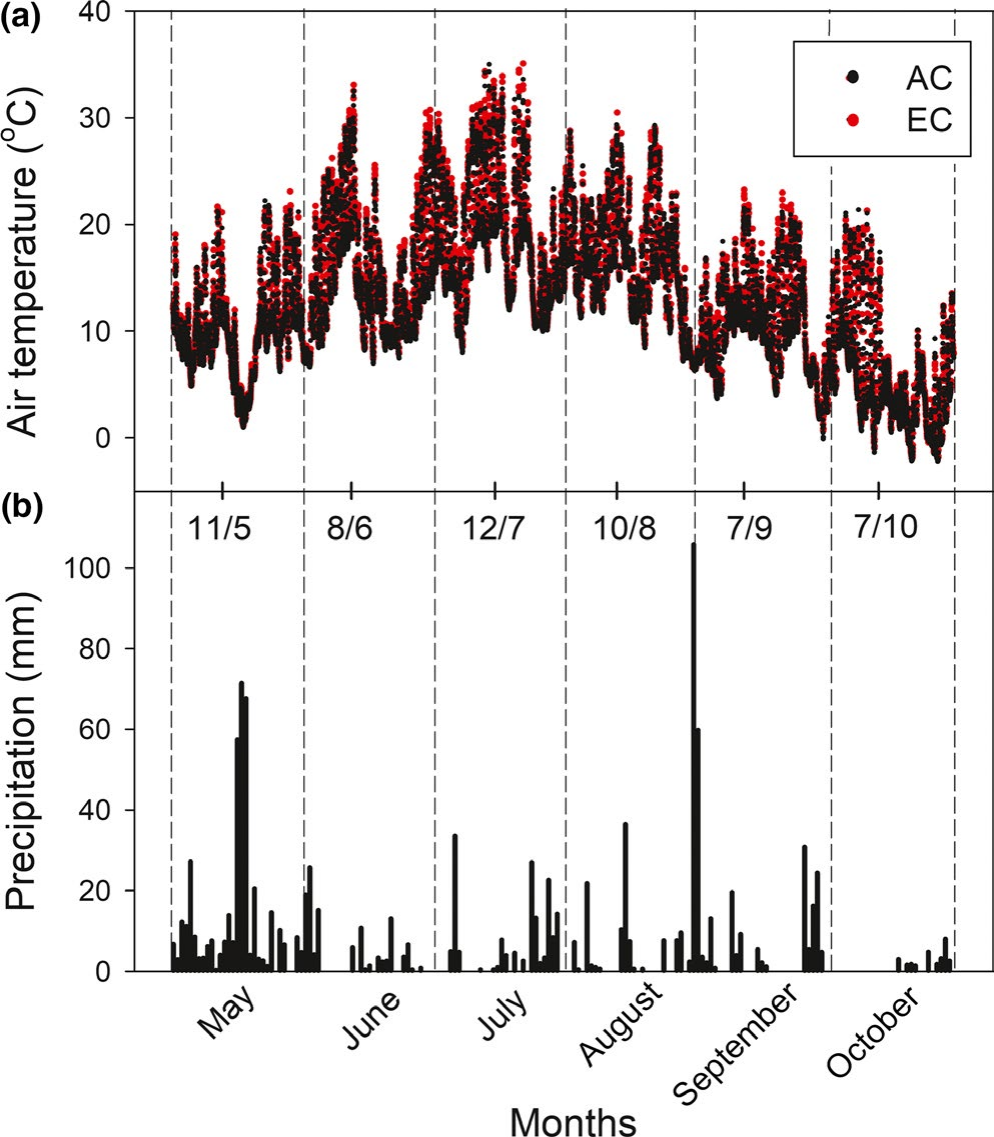
Seasonal course of air temperature (a) measured 2 m above the ground in the glass domes, maintained at ambient and elevated [CO_2_], and the sum of daily precipitation (b) during the growing season 2010 (May–October). Dates indicate the days of physiological measurements

**Figure 2:**
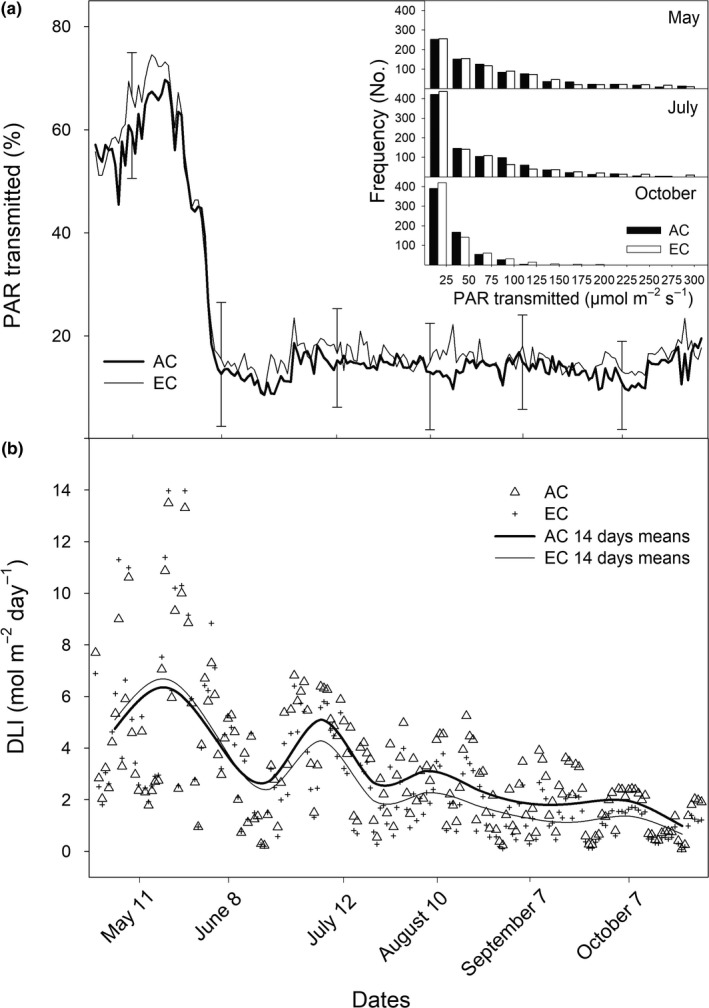
Relative (a) and cumulative—daily light integral (DLI; b) amounts of photosynthetically active radiation (PAR) transmitted through forest canopy under ambient (AC) and elevated (EC) [CO_2_] recorded during growing season 2010 (May–October). Transmittance and DLI values were calculated from 30‐min averages of PAR. Error bars represent standard deviations estimated on sampling dates. The frequency of nonzero PAR in AC and EC understories is shown for three selected months (inset plot)

The site is characterized by a mean annual temperature of 6.7 ± 1.1°C and precipitation of 1,316 ± 207 mm (average ± standard deviation for the period 1998–2010). The year 2010, in which the measurements were made, was characterized by a mean annual temperature of 6.0°C, the maximal air temperatures in July (35°C), and an annual precipitation of 1,297 mm with the highest amounts of precipitation in mid‐May and at the end of August and early September (Figure 1). Light penetration into the tree understorey amounted to 80% before leaf development (May), while it was only 20% during the peak of the growing season (July–September). The daily maxima of photosynthetically active radiation (PAR) in the forest understorey amounted up to 300 µmol m^−2^ s^−1^ in May, but were only 175 µmol m^−2^ s^−1^ in October (Figure 2a). Daily light integral (DLI; Figure 2b), mean half‐hour PAR values integrated over a day, ranged from 0.1 mol m^−2^ day^−1^ (cloudy sky autumn days) up to 14 mol m^−2^ day^−1^ (clear sky spring days).

### 2.2 Gas exchange measurements

Seasonal courses (May 11–12, June 8–9, July 12–14, August 10–11, September 7–8, and October 7–8, 2010) of gas exchange parameters were measured on fully developed leaves during the extended noon hours (11:00–15:00). An open infrared gas analyser Li‐6400 (Li‐Cor) was used to measure the relationship between the CO_2_ assimilation rate (*A*) and intercellular CO_2_ concentration (*C*i). The *A/C*i response curves were produced at saturating light intensity (1,200 µmol m^−2^ s^−1^) and the following [CO_2_] in the leaf assimilation chamber: 1,500, 1,100, 700, 385, 250, 100, and 50 µmol CO_2_/mol. Such range of [CO_2_] enabled the modeling of the both parts of *A/C*i curves limited by Rubisco activity and electron transport rate (Figure S2). The measured leaves were kept at constant temperature and vapor pressure deficit corresponding to the natural seasonal variability (Figure 1). A biochemical model of photosynthesis (von Caemmerer, 2000) was applied to derive the maximum in vivo Rubisco carboxylation rate (*V*_Cmax_) and maximum electron transport (*J*_max_) from the *A*/*C*i response curves using Photosyn Assistant software (Dundee Scientific). To model the seasonal temperature effects on Michaelis–Menten constants of Rubisco for carboxylation and oxygenation, the approach of Harley, Thomas, Reynolds, and Strain (1992) was applied. Subsequently, the temperature functions proposed by Bernacchi, Singsaas, Pimentel, Portis, and Long (2001) were used to normalize *V*_Cmax_ and *J*_max_ values to 25°C.

The relationship between *A* and PAR (*A*/PAR) was obtained at growth [CO_2_], that is, at 385 μmol CO_2_/mol for AC plants and at 700 μmol CO_2_/mol for EC plants. The PAR used was 0, 25, 50, 100, 200, 400, 800, and 1,200 μmol m^−2^ s^−1^. For each measurement, leaf temperature and relative air humidity inside the assimilation chamber were kept stable on the average values of the previous 3 days (15–25°C and 45%–65%). Dark respiration rate of leaves (*R*_D_) was estimated after 15 min of darkening. Instantaneous rates of *A* (Figure S3) were subsequently modeled as a nonrectangular hyperbolic function of incident PAR using a Nelder–Mead algorithm (Urban et al., 2007) to determine values of apparent quantum efficiency (AQE), light compensation irradiance (LCI), and light saturation estimate (LSE). In addition, *A* values at a PAR of 50 (*A*_50_), 200 (*A*_200_), and 1,200 μmol m^−2^ s^−1^ (*A*_max_), representing the most frequent and maximum PAR in the understorey, respectively, were calculated. Intrinsic water use efficiency was defined as the ratio of CO_2_ assimilation rate to stomatal conductance at a PAR of 50 (iWUE_50_ = *A*_50_/*G*_S50_) and 1,200 μmol m^−2^ s^−1^ (iWUE_max_ = *A*_max_/*G*_Smax_). Carbon ratio, a proxy of carbon balance, was subsequently calculated as *A*_200_/*R*_D_.

### 2.3 Morphological and production parameters

Fully developed leaves of *C. arundinacea* and *L. sylvatica*, on which the physiological measurements were carried out, were sampled throughout the growing season (May–October) to analyze their dry mass and leaf area. The leaf area was determined by a leaf area meter LI‐3000A (Li‐Cor) and subsequently dried to constant mass at 60°C for 48 hr. In addition, a destructive sampling of total above‐ and belowground biomass of five plants of both grass species was performed in August 2010. Plant parts were dried to constant mass at 60°C for 48 hr. Leaf mass area (LMA; leaf dry mass per leaf area) and the ratio between root and shoot mass (R/S) were calculated.

### 2.4 Statistical analyses

**Figure 3:**
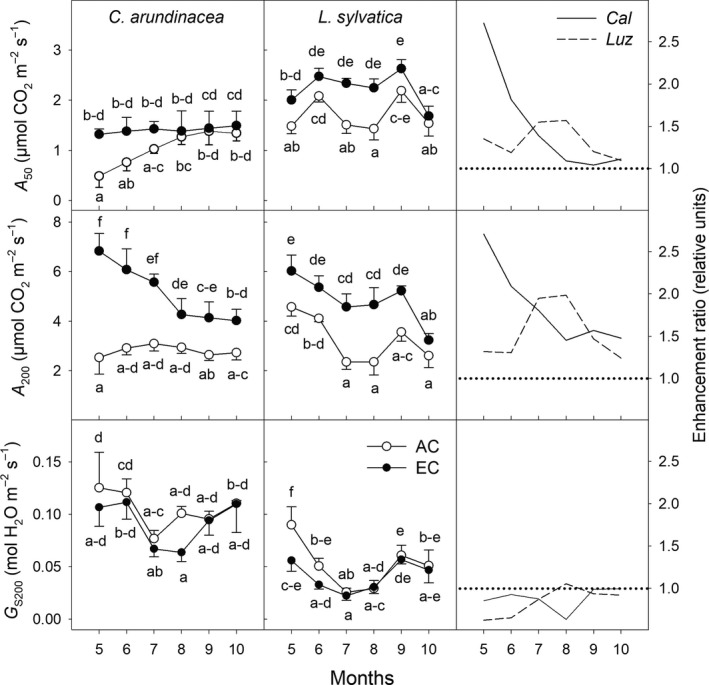
Seasonal courses of CO_2_ assimilation rate (*A*) estimated at growth [CO_2_], and photosynthetically active radiation (PAR) of 50 (*A*_50_) and 200 µmol m^−2^ s^−1^ (*A*_200_) and stomatal conductance at a PAR of 200 µmol m^−2^ s^−1^ (*G*_S200_) in *Calamagrostis arundinacea* (*Cal*) and *Luzula sylvatica* (*Luz*) developed in the understorey. The measurements were made during the fourth growing season (May–October, 2010) of cultivation under ambient (AC) and elevated [CO_2_] (EC). Mean values (symbols) and standard deviations (error bars) are presented in the figure. Different letters denote significantly different values within each species separately (Fisher's LSD test *p* ≤ .05 after ANOVA); *n* = 5. Enhancement ratio is equal to the ratio of the parameter estimated under EC and AC conditions

**Figure 4:**
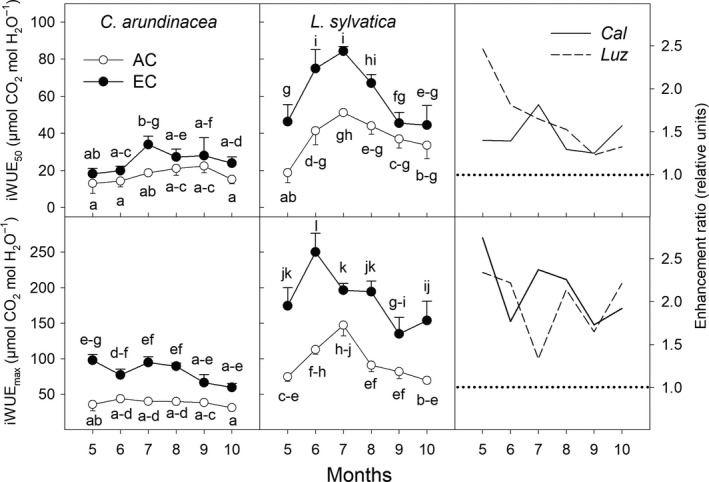
Seasonal courses of intrinsic water use effciency estimated at growth [CO_2_] and conditions of low (50 µmol m^−2^ s^−1^; iWUE_50_) and high PAR intensity (1,200 µmol m^−2^ s^−1^; iWUE_max_) in *Calamagrostis arundinacea* (*Cal*) and *Luzula sylvatica* (*Luz*) developed in forest understory. The measurements were done during the fourth growing season (May–October, 2010) of cultivation under ambient (AC) and elevated [CO_2_] (EC). Mean values (symbols) and standard deviations (error bars) are presented in the figure. Different letters denote significantly different values within each species separately (LSD test *p* ≤ .05 after ANOVA); *n* = 5. Enhancement ratio is equal to the ratio of the parameter estimated under EC and AC growing conditions

**Figure 5:**
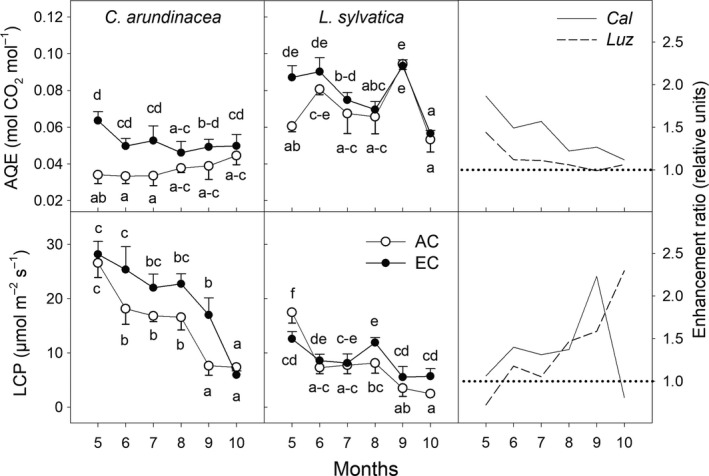
Seasonal courses of photosynthetic parameters derived from the relationship of CO_2_ assimilation rate and photosynthetically active radiation: apparent quantum efficiency (AQE) and light compensation point (LCP) *Calamagrostis arundinacea* (*Cal*) and *Luzula sylvatica* (*Luz*) developed in forest understorey. The measurements at growth [CO_2_] were made during the fourth growing season (May–October 2010) of cultivation in ambient (AC) and elevated [CO_2_] (EC). Mean values (symbols) and standard deviations (error bars) are presented in the figure. Different letters denote significantly different values separately for each species (LSD test *p* ≤ .05 after ANOVA); *n* = 5. Enhancement ratio is equal to the ratio of the parameter estimated under EC and AC conditions

**Figure 6:**
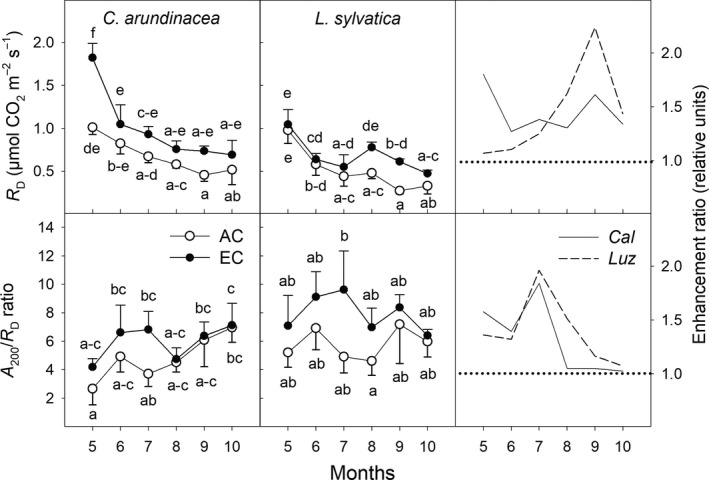
Seasonal courses of dark respiration rate (*R*_D_) and *A*_200_/*R*_D_ ratio estimated in leaves of *Calamagrostis arundinacea* (*Cal*) and *Luzula sylvatica* (*Luz*) developed in a forest understorey at ambient (AC) and elevated [CO_2_] (EC). The gas exchange measurements were done at growth [CO_2_] during the fourth growing season (May–October, 2010) of cultivation in AC and EC conditions. Mean values (symbols) and standard deviations (error bars) are presented. Different letters denote significantly different values separately for each species (LSD test *p* ≤ .05 after ANOVA); *n* = 5. Enhancement ratio is equal to the ratio of the parameter estimated under EC and AC conditions

**Figure 7:**
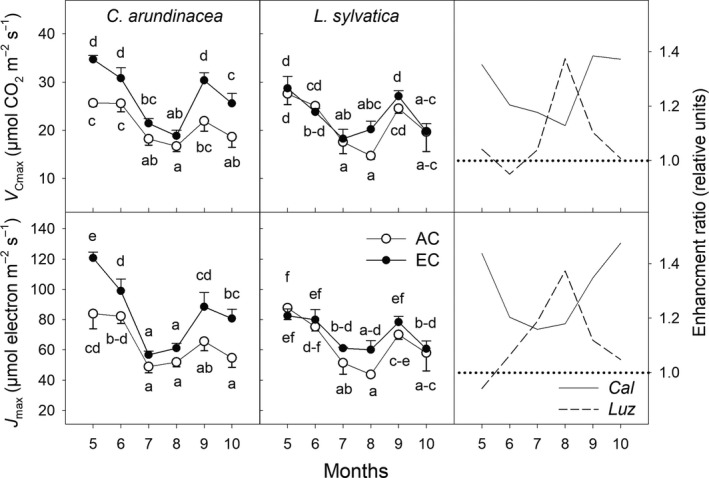
Seasonal courses of light‐saturated rate of in vivo Rubisco carboxylation (*V_C_*_max_) and light‐saturated rate of electron transport (*J*_max_) estimated at a reference temperature of 25°C in leaves of *Calamagrostis arundinacea* (*Cal*) and *Luzula sylvatica* (*Luz*) developed in a forest understorey at ambient (AC) and elevated [CO_2_] (EC). The measurements were done during the fourth growing season (May–October, 2010) of cultivation in AC and EC conditions. Mean values (symbols) and standard deviations (error bars) are presented. Different letters denote significantly different values within each species separately (LSD test *p* ≤ .05 after ANOVA); *n* = 5. Enhancement ratio is equal to the ratio of the parameter estimated under EC and AC conditions

**Figure 8:**
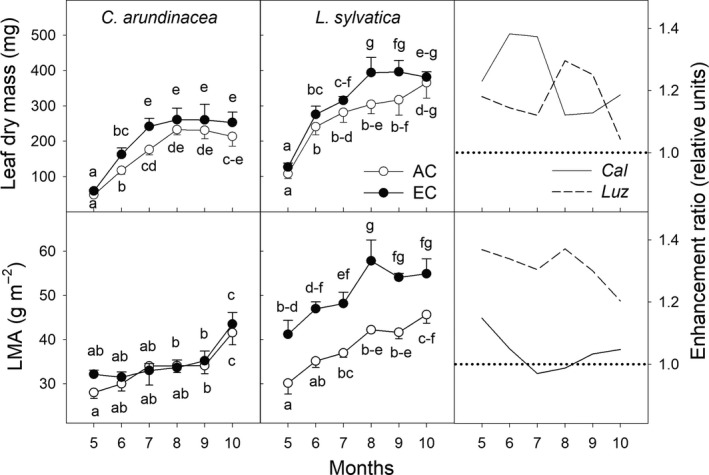
Seasonal courses of leaf dry mass and leaf mass per area (LMA) of *Calamagrostis arundinacea* (*Cal*) and *Luzula sylvatica* (*Luz*) developed in forest understorey at ambient (AC) and elevated [CO_2_] (EC). The measurements were done during the fourth growing season (May–October 2010) of cultivation in AC and EC conditions. Mean values (symbols) and standard deviations (error bars) are presented. Different letters denote significantly different values within each species separately (LSD test *p* ≤ .05 after ANOVA); *n* = 10. Enhancement ratio is equal to the ratio of the parameter estimated under EC and AC conditions

The data were evaluated by means of an analysis of variance, using the statistical package STATISTICA 12 (StatSoft). Three‐way ANOVA analysis was used to test the effect of species (*C. arundinacea* vs. *L. sylvatica*), [CO_2_] (AC vs. EC), and date within the season (measuring dates during the whole growing season) on morphological and physiological parameters. Two‐way ANOVA analysis was subsequently used to test seasonal differences between means and the effect of [CO_2_] on morphological and physiological parameters in each plant species separately (Figures 3, 4, 5, 6, 7). The Fisher's LSD post‐hoc test was used to evaluate differences between means. For the destructive analysis of above‐ and belowground biomass, the differences between means were tested using one‐sample *t* tests. Significance levels are reported in the Figure 8 and tables as a significant with *p ≤ .05, **p ≤ .01, and ***p ≤ .001.

## 3 RESULTS

The three‐way ANOVA of the whole dataset (two species, two [CO_2_], and six measuring campaigns along the growing season) showed a significant effect of species on all photosynthetic parameters except *A* at PAR 200 μmol m^−2^ s^−1^ (*A*_200_). Species had a significant effect on aboveground morphological parameters, while belowground biomass did not differ significantly between species. Significant effects of [CO_2_] on all photosynthetic parameters, leaf DM, leaf mass area, root DM, and R/S ratio were observed (Table 2). The effect of time, that is, seasonal dynamics, was significant in all observed photosynthetic and morphological parameters.

**Table 2:** The effects of species (Sp), CO_2_ concentration ([CO_2_]), time (*T*), and their mutual interactions (×) on photosynthetic and morphological parameters: CO_2_ assimilation rate (*A*) estimated at growth [CO_2_] and a photosynthetically active radiation (PAR) of 50 (*A*_50_), 200 (*A*_200_), and 1,200 µmol m^−2^ s^−1^ (*A*_max_), apparent quantum efficiency (AQE), light compensation point (LCP), light saturation estimate (LSE), stomatal conductance at a PAR 200 µmol m^−2^ s^−1^ (*G*_S200_), dark respiration rate (*R*_D_), maximum rate of *in vivo* Rubisco carboxylation (*V_C_*_max_) and maximum rate of electron transport (*J*_max_) estimated at a reference temperature of 25°C, intrinsic water use effciency estimated at growth [CO_2_] and conditions of low (50 µmol m^−2^ s^−1^; iWUE_50_) and high PAR intensity (1,200 µmol m^−2^ s^−1^; iWUE_max_), carbon ratio (*A*_200_/*R*_D_), leaf dry mass (Leaf DM), leaf mass per area ratio (LMA), shoot (root) dry mass (Shoot DM, Root DM), and root‐to‐shoot ratio (R/S)

Effect	Sp	[CO_2_]	*T*	Sp × [CO_2_]	Sp × *T*	[CO_2_] × *T*	Sp × [CO_2_] × *T*
*df*	1	1	5	1	5	5	5
*A*_50_	65.0***	31.6***	3.3**	0.2^n.s.^	4.6***	0.5^n.s.^	1.4^n.s.^
*A*_200_	0.1^n.s.^	98.7***	7.9***	3.7^n.s.^	2.1^n.s.^	2.0^n.s.^	2.1^n.s.^
*A*_max_	127.8***	181.3***	33.5***	25.2***	5.1***	4.0**	6.1***
AQE	166.5***	24.7***	5.1***	2.0^n.s.^	6.9***	2.5*	0.1^n.s.^
LCP	136.7***	11.8***	30.4***	5.1*	4.0**	1.9^n.s.^	1.2^n.s.^
LSE	364.6***	49.3***	15.3***	15.5***	7.8***	3.3*	1.6^n.s.^
*G*_S200_	97.3***	4.7*	6.8***	0.1^n.s.^	1.1^n.s.^	0.5^n.s.^	0.6^n.s.^
*R*_D_	25.4***	26.6***	20.4***	2.5^n.s.^	1.3^n.s.^	0.9^n.s.^	1.8^n.s.^
*V*_Cmax_	6.1*	24.9***	27.1***	9.0**	0.5^n.s.^	0.7^n.s.^	1.4^n.s.^
*J*_max_	8.3**	27.9***	29.1***	7.9**	1.5^n.s.^	0.2^n.s.^	2.2^n.s.^
iWUE_50_	109.1***	33.0***	5.9***	8.0**	3.3**	0.9^n.s.^	0.7^n.s.^
iWUE_max_	204.7***	134.5***	7.2***	16.0***	4.0**	1.8^n.s.^	1.8^n.s.^
*A*_200_/*R*_D_	5.8*	7.2**	1.6^n.s.^	0.6^n.s.^	0.7^n.s.^	0.7^n.s.^	0.1^n.s.^
Leaf DM	119.9***	17.9***	61.0***	0.1^n.s.^	1.3^n.s.^	0.5^n.s.^	0.6^n.s.^
LMA	101.3***	40.6***	14.4***	27.0***	2.5*	0.2^n.s.^	0.5^n.s.^
Shoot DM	6.4*	1.1^n.s.^	—	0.1^n.s.^	—	—	—
Root DM	2.1^n.s.^	6.5*	—	0.01^n.s.^	—	—	—
R/S	0.1^n.s.^	15.0**	—	0.5^n.s.^	—	—	—

Note

Results of three‐way ANOVA (*df*, *F*‐value) analyses are shown (n.s., non significant; *p ≤ .05; **p ≤ .01; ***p ≤ .001). Two‐way ANOVA was used to analyze shoot DM, root DM, and R/S values.

We found a significant species × [CO_2_] interactive effect on photosynthetic parameters estimated under high light intensities (*A*_max_, LSE, *V*_Cmax_, and *J*_max_), but not on the photosynthetic parameters derived at low PAR (*A*_50_, *A*_200_, *G*_S200_, AQE, *R*_D_, and *A*_200_/*R*_D_ ratio) and parameters of biomass production (shoot and leaf DM, and R/S ratio). Also [CO_2_] and time had a significant interactive effect on some photosynthetic parameters; however, [CO_2_] × time interaction was not as robust as compared to species × [CO_2_]. The only significant effect of species × [CO_2_] × time was found for *A*_max_ expressed per unit leaf area.

### 3.1 Photosynthetic parameters

Leaves of the shade‐tolerant *L. sylvatica* had generally higher values of *A*_50_ (CO_2_ assimilation rate at 50 µmol m^−2^ s^−1^) under AC conditions as compared to leaves of the less shade‐tolerant *C. arundinacea* (Figure 3). Stomata of *L. sylvatica* plants were, however, more sensitive to summer (July–August) drought conditions than stomata of *C. arundinacea* plants irrespective of [CO_2_] treatment. Reduced stomatal conductance (*G*_S_) subsequently led to a substantial reduction of *A*_50_ as well as *A*_200_ values under AC conditions as compared to spring months, but this negative effect of reduced *G*_S_ on *A* was compensated by EC (Figure 3).

Throughout the growing season the EC led to an increase of *A*_max_ in *C. arundinacea* by 74%–150%, while the [CO_2_]‐stimulation amounted only to 23%–82% in *L. sylvatica* (Figure S4). The EC conditions also stimulated the *A* values at low PAR (*A*_50_ and *A*_200_; Figure 3) in both grass species. The seasonal course of photosynthetic acclimation to EC was, however, species‐specific. While EC conditions led to increases of *A*_50_ and *A*_200_ in *C. arundinacea*, particularly at the beginning of the growing season (May–June), the highest and statistically significant stimulation of *A*_50_ and *A*_200_ in *L. sylvatica* was observed during July and August when the lowest *G*_S_ values were recorded (Figure 3).

Generally, strong shade‐tolerant *L. sylvatica* had higher iWUE under both [CO_2_] treatments as compared to sun‐demanding *C. arundinacea* (Figure 4). Elevated [CO_2_] increased iWUE in the both grass species studied. This increase was approximately 100% for the most of the growing season at saturating light conditions (iWUE_max_), while it amounted only to 50% under low light intensities (iWUE_50_).

Leaves of *L. sylvatica* plants had higher AQE and lower LCP than *C. arundinacea* under both [CO_2_] conditions, throughout the whole growing season. A significant stimulatory effect of EC on AQE was found in both species at the beginning of the growing season, but it gradually diminished, particularly in *L. sylvatica* (Figure 5). The LCP values significantly decreased throughout the growing season in both growth environments. Although EC led to an increase in LCP of up to 125% and 130% in *C. arundinacea* and *L. sylvatica*, respectively, these differences were mostly statistically nonsignificant (*p* > .05; Figure 5). On the contrary, a highly significant positive effect of the EC treatment on the light saturation estimate (LSE) was found in both species (Figure S4), except at the beginning (May) and end of the growing season (October).

Leaf dark respiration (*R*_D_) tended to decrease throughout the growing season in both grass species and [CO_2_] treatments studied. While EC stimulated RD values in *C. arundinacea* plants at the beginning of the growing season (May), significant stimulation of *R*_D_ by EC conditions was found during August and September in *L. sylvatica* plants (Figure 6). Carbon ratio, the ratio between *A*_200_ and *R*_D_, was substantially stimulated be EC amounting up to 170%–190% in July; however, these differences were statistically not significant (*p* > .05). Moreover, the EC stimulation of the *A*_200_/*R*_D_ ratio diminished in August in *C. arundinacea* and in September in the *L. sylvatica* plants (Figure 6).

*V*_Cmax_ and *J*_max_ reached the lowest values during July and August (Figure 7), that is, the months when the highest temperature and the lowest total precipitation were measured (Figure 1). The EC conditions led to a significant stimulation of *V*_Cmax_ and *J*_max_ in *C. arundinacea* at the beginning (May, June) and end of the growing season (September, October), but in *L. sylvatica* the stimulation occurred during the summer months with a peak in August.

### 3.2 Morphological and production parameters

In both grass species, leaf dry mass increased under EC as compared to AC, however, only significantly in July for *C. arundinacea* and in August for *L. sylvatica*. While no significant differences in leaf mass per area (LMA) were found in *C. arundinacea*, a significant increase in LMA, in response to the EC treatment, was observed in *L. sylvatica* during the whole experimental period, except in October (Figure 8).

**Figure 9:**
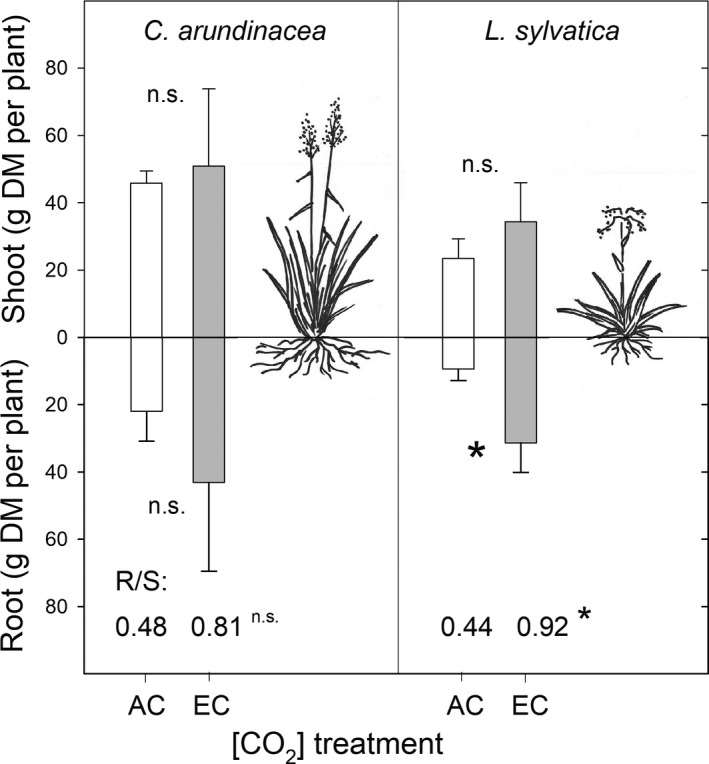
Mean values (columns) of shoot and root dry mass and root‐to‐shoot ratio (R/S) of *Calamagrostis arundinacea* and *Luzula sylvatica* developed in forest understorey at ambient (AC) and elevated [CO_2_] (EC). The sampling was done in September 2010, that is, after 4 years of cultivation in AC and EC conditions. Error bars represent standard deviations. A *t* test was performed to compare differences between means of AC and EC treatments within individual plant species (n.s., non significant; *p ≤ .05; *n* = 5)

Destructive sampling of experimental plants in August showed significant effects of species on shoot dry mass. While no significant response to EC in dry mass accumulation was found in *C. arundinacea*, a marked increase in root dry mass was observed in *L. sylvatica* (Figure 9). This response led to a significant increase of the R/S ratio in *L. sylvatica* under EC (0.92) in comparison with AC (0.44) growing conditions.

## 4 DISCUSSION

Climate change may lead to an increase of light intensity in forest understories due to triggered tree die‐off and reduction of overstorey canopy (Royer et al., 2011) as well as its reduction when the overstorey leaf area is stimulated by EC conditions (Norby et al., 2005). The contribution of understorey vegetation to carbon sequestration and other ecosystem functions can be relatively high under both radiation conditions if the understorey vegetation shows sufficient plasticity for acclimation (Nilsson & Wardle, 2005).

Within this study, we tested the hypothesis that elevated [CO_2_] stimulates photosynthesis and growth of understorey plants species even under low light intensities and that understorey species with different dynamics of development and light requirements can utilize different light niches during the vegetation season to profit from elevated [CO_2_]. To understand the mechanisms of acclimation plasticity of understorey plants, we studied seasonal dynamics in photosynthetic responses of two distinct grass species—less shade‐tolerant *C. arundinacea* with rapid transition to generative stage and highly shade‐tolerant *L. sylvatica* with slow development.

### 4.1 [CO_2_] stimulation of photosynthesis at low light intensity

In general, the stimulatory effect of elevated [CO_2_] on photosynthetic assimilation varies depending on the functional group and interactions with other environmental conditions. Ainsworth and Rogers (2007) concluded that trees are more responsive to elevated [CO_2_] than other functional groups, including herbaceous understorey species. These conclusions are, however, mainly based on studies where the plants were exposed to high light intensities, while studies conducted on shade‐acclimated leaves and understorey vegetation received little attention (Kim, Oren, & Qian, 2016; Valladares, Laanisto, Niinemets, & Zavala, 2016). In the present study, we found evidences supporting the hypothesis that EC substantially stimulates photosynthesis (Figure 3) and partially also the growth (Figures 8 and 9) of understorey plants naturally exposed to low DLIs (0.1–14 mol m^−2^ day^−1^), that is, conditions when photosynthesis is limited particularly by an insufficient rate of electron transport and formation of electrochemical potential on thylakoid membrane (Farquhar et al., 1980; von Caemmerer, 2000). However, the analysis of photosynthetic light curves (Figure S2) shows considerable species‐specific differences in EC stimulation in response to light intensity. While in shade‐tolerant species *L. sylvatica* changes the relative stimulation by EC only little with light intensity, less tolerant species *C. arundinacea* shows a significant increase of relative stimulation by EC with light intensity. In addition, photosynthetic stimulation by EC shows in *C. arundinacea* changes with decreasing role of light intensity during the vegetation season. One of the main reasons for maintaining relatively high stimulation by EC under low light intensities in understorey vegetation is reduced downregulation of photosynthesis which is driven by accumulation of carbohydrates and mediated by hexokinase signaling pathway (Kelly et al., 2013). This signaling pathway senses the imbalance between carbon source and carbon sinks. Higher light intensities lead in shade‐tolerant species to rapid predominance of carbon source above carbon sink and subsequent feedback regulation of photosynthesis. On the contrary, shade‐intolerant species provide sufficient carbon sinks even under high light intensities, which means that downregulation of photosynthesis occurs only at high light intensities (Springer & Thomas, 2007). As the carbon sink strongly depends on plant development stage, with the highest sink during rapid vegetative growth, the downregulation of photosynthesis can also explain the seasonal changes in EC stimulation. Carbon sink capacity may be further modulated by nitrogen and water availability, thus altering the response to EC (Leakey et al., 2009).

In agreement with previous studies (summarized in Kim et al., 2016), we have found greater [CO_2_] effect on *A*_max_ (Figure S4) and smaller enhancement of *A*_50_ (Figure 3) and AQE (Figure 5). However, the effect of EC changed asynchronously with light intensity for individual species, with less growth of EC stimulation above PAR intensities 200 μmol m^−2^ s^−1^ in *L. sylvatica*. Consistent stimulation of *A* and AQE by elevated [CO_2_] at low light intensities was found also in tropical understorey vegetation (Hättenschwiler & Körner, 1996, 2000; Würth et al., 1998) and shade‐acclimated shoots of *P. abies* (Marek et al., 2002). Besides role of carbon source and sink balance and limited feedback regulation of photosynthesis in understorey vegetation, such enhancements are also likely caused by a reduced photorespiration rate due to an increased ratio of intercellular [CO_2_] to [O_2_] (Drake et al., 1997; Farquhar et al., 1980; Way et al., 2015).

*V*_Cmax_ and *J*_max_ parameters characterizing biochemical limitations of photosynthesis represent important indicators of photosynthetic downregulation. In our study, EC had a slight positive effect on the both *V*_Cmax_ and *J*_max_ values, indicating no occurrence of photosynthetic downregulation in both species, although the response of both parameters to EC changed during vegetation season and showed species‐specific temporal dynamics (higher stimulation in summer for *L. sylvatica* and in spring and autumn for *C. arundinacea*; Figure 7).

In contrary to DeLucia and Thomas (2000), who observed the proportionately greater stimulation of *J*_max_ by [CO_2_], *V*_Cmax_ to *J*_max_ ratio remained constant in our study with two understorey grass species. Noticeably, both overstorey tree species, *P. abies* and *Fagus sylvatica*, had lower *V*_Cmax_ and *J*_max_ values under EC than AC conditions indicating photosynthetic downregulation (Košvancová et al., 2009). It can be assumed that an enhanced accumulation of nonstructural carbohydrates, particularly hexoses, under EC conditions, is at low DLIs of understorey plants insufficient to initiate a feedback inhibition of photosynthesis including among others a shortage of inorganic phosphate in the chloroplast for ATP synthesis and RuBP regeneration, a repression of the expression of genes transcribing for Rubisco and/or a reduction of Rubisco content and activity (reviewed in Ceulemans & Mousseau, 1994; Leakey et al., 2009; Urban, 2003; Way et al., 2015). Moreover, it seems that C sink strength is not reduced in understorey plants as documented by positive [CO_2_] effect on the growth of aboveground and belowground biomass (Figures 8 and 9). However, it should be emphasized that the degree of [CO_2_]‐induced enhancement of growth may be strongly reduced under the conditions of insufficient nutrient, particularly nitrogen, availability (Kim et al., 2016).

### 4.2 Responses to elevated [CO_2_] are species‐specific

To test the hypothesis that species differing in shade‐tolerance also have a different sensitivity to EC, *L. sylvatica* and *C. arundinacea* were investigated in this study. Higher values of *A*_50_ and AQE together with lower LCP in *L. sylvatica* than *C. arundinacea* under AC conditions (Figures 3 and 5) confirmed that *L. sylvatica* is a more shade‐tolerant species than *C. arundinacea*. We found that EC conditions substantially stimulate the formation of above‐ and particularly belowground biomass of shade‐tolerant *L. sylvatica*, while only insignificant increases were observed in *C. arundinacea* plants (Figure 9). This is in accordance with a higher [CO_2_] stimulation of *A*_50_, *A*_200_, and *A*/*R*_D_ ratio in *L. sylvatica* than in *C. arundinacea*, particularly in summer months. Also Kubiske and Pregitzer (1996) concluded an increasing stimulation effect of elevated [CO_2_] on photosynthetic parameters with an increasing shade‐tolerance of plant species. In contrary, Hättenschwiler (2001) observed high variability of physiological and morphological responses to elevated [CO_2_] in five tree species of forest understorey even across the narrow range of successional status and shade‐tolerance of the species studied. Our results show strong seasonality in species responses to EC and imply that differences in EC stimulation are controlled by plant development modulating sink capacity. While *C. arundinacea* transits to generative stage after short period of fast vegetative growth inducing thus senescence of older leaves, *L. sylvatica* is typical by continuous vegetative growth over the whole vegetation season. Integration of EC stimulation over the whole vegetation season thus results in higher biomass EC stimulation in *L. sylvatica*.

It is hypothesized that the physiological mechanism behind the stimulatory effect of elevated [CO_2_] on carbon gain under low light intensities includes an increase of AQE and a reduction of LCP (Osborne et al., 1998). While our study confirmed higher AQE values under EC conditions and particularly in *C. arundinacea* at the beginning of the vegetation season, the hypothesis of reduced LCP was not supported by our data (Figure 5). In accordance with DeLucia and Thomas (2000), we have found that EC conditions led to higher LCP values, that is, higher light intensities are required to compensate between assimilatory and respiratory processes, particularly in the less shade‐tolerant *C. arundinacea* plants. Such increase in LCP values is caused by the increase in *R*_D_ under elevated [CO_2_] (Figure 6) leading to an overall shift of the *A*/PAR curves (Figure S3). Accordingly, we conclude that increased carbon uptake in understorey plants under EC conditions is primarily caused by increased AQE, that is, reduced photorespiration rate.

[CO_2_]‐induced changes in biomass partitioning between shoots (S) and roots (R) also seems to be species‐specific. Although both grass species showed an increase in root biomass and an increase in R/S ratio under EC conditions, these changes were significant only in the shade‐tolerant *L. sylvatica* (Figure 9). Arnone et al. (2000) studied the response of root systems to elevated [CO_2_] in intact native grassland ecosystems and found one group of plants with no change in the root systems, and the second group with growth increases of 38% in average. Increased root production under elevated [CO_2_] could, however, be followed by increased root mortality and decomposition rates which may lead to only small changes in root biomass, particularly in high soil moisture conditions (Pendall, Osanai, Williams, & Hovenden, 2003). Differences in root growth stimulation under EC conditions can be explained by variety of mechanisms among which nutrient availability (especially nitrogen) plays a crucial role. Since the carbon investment into the root system is energetically disadvantageous, the plants increase the root system in response to EC only under nitrogen limiting conditions together with improved nutrient uptake by mycorrhiza (Arnone et al., 2000).

More pronounced growth stimulation of shade‐tolerant species by elevated [CO_2_] was confirmed in a meta‐analysis by Kerstiens (2001). However, differences between shade‐tolerant and shade‐intolerant species only occurred at high DLIs (Poorter et al., 2019). Kerstiens (1998) suggested that shade‐tolerance as such does not play a role in response to elevated [CO_2_], but that functional traits associated with the ability to survive suppression of growth in the forest understorey are crucial for growth response to elevated [CO_2_] (e.g., ability to harvest light, water, and nutrients). Highest responses to elevated [CO_2_] were thus found in species with generally low relative growth rate, low leaf nitrogen content, and high R/S ratio and LMA (Kerstiens, 2001). These are typical traits for *L. sylvatica*, which showed higher growth stimulation by EC, particularly in summer months with a closed canopy, but slightly increasing DLIs given by longer days and higher incident PAR above the canopy.

### 4.3 Seasonality of responses to elevated [CO_2_]

Pronounced seasonal pattern in *V*_Cmax_ and *J*_max_ was observed in both understorey grass species studied. In accordance with the study by Xu and Baldocchi (2003), maximum values of *V*_Cmax_ and *J*_max_ were recorded in spring after leaf expansion followed by minimal values during hot and dry summer months and partial recovery at the end of summer and autumn. Such seasonal patterns were found under the both [CO_2_] treatments (Figure 7).

Our results also support the hypothesis that the stimulatory effect of EC is changing throughout the growing season and is based on species‐specific differences in shade‐tolerance and developmental dynamics, allowing the two species to exploit different light niches during the season. The existence of two light niches in early spring and during the summer months exploited by typically sun‐demanding and shade‐tolerant understorey vegetation, respectively, has been proved in our experimental mixed forest (Figure 2b).

For *C. arundinacea*, the EC conditions led to an increase in *A*_50_ (the most frequent light intensity of a forest understorey; Figure 3) and *A*_200_/*R*_D_ ratio (proxy to carbon balance of leaves; Figure 6), particularly at the beginning of the growing season when the leaf area of the overstorey trees was not fully developed. On the other hand, these parameters were substantially stimulated in *L. sylvatica* by EC in the summer months (July–August) which can be attributed to significantly lower light saturation intensities, compared to *C. arundinacea*, and better utilization of low intensities during longer days. Pronounced stimulation by EC in the summer months can also be associated with lower water availability, which was confirmed by reduced stomatal conductance (Figure 3). The EC generally increases iWUE (Figure 4) and may thus reduce the negative impact of limited water availability (Valladares et al., 2016). Therefore, not only the tolerance to shade conditions, but also the sensitivity of plants to other environmental perturbations, like drought, may further modulate the final response of understorey plants to EC and its seasonal dynamics. This is in agreement with the findings by Belote et al. (2004) that responses of understorey plants to elevated [CO_2_] are mediated by soil water availability. Several other studies also concluded a positive effect of elevated [CO_2_] on CO_2_ assimilation rate, plant water relations, and growth, during drought or water‐limited periods (Ainsworth & Long, 2005; Guehl, Picon, Aussenac, & Gross, 1994; Tschaplinski et al., 1995). In our study, however, the relatively even distribution of precipitation in July and August suggests that peak stimulation by EC during these months was more related to species‐specific differences.

Seasonal changes in the gas exchange parameters were in accordance with the seasonal dynamics of leaf dry mass, LMA, and their enhancement ratios (Figure 8). Based on a meta‐analysis, Poorter and Navas (2003) concluded that elevated [CO_2_] increases LMA in almost all C3 plants. However, we have observed this increase significant in shade‐tolerant *L. sylvatica*, but not in sun‐demanding *C. arundinacea*. The causes of negligible EC effect on LMA in *C. arundinacea* can be twofold. First, the photosynthetic stimulation in this species was observed only during rapid vegetative growth with high sink for carbon represented by newly developing leaves. Translocation of carbohydrates to new leaves thus limited the direct effect carbohydrate accumulation on LMA. Second, the low effect of EC on LMA in *C. arundinacea* could be explained by higher production of flowering shoots in comparison with AC in this species (data not shown) and thus lower biomass allocation to vegetative leaves during flowering. Also Jablonski, Wang, and Curtis (2002) reported significantly enhanced number of flowers and seeds in plants grown under EC in comparison with AC.

## 5 CONCLUSIONS

Our data support the hypothesis that elevated [CO_2_] increases photosynthetic carbon uptake and stimulates the growth of understorey plant communities. In addition, we confirmed the hypothesis that species with distinct dynamics of development and shade‐tolerance utilize different light niches during vegetation season to profit from rising [CO_2_]. In our study, the elevated [CO_2_] stimulated particularly growth of shade‐tolerant *L. sylvatica* that was able to sustain [CO_2_]‐stimulated photosynthesis at natural light of low intensity during much of the growing season. In contrary, such [CO_2_]‐stimulated photosynthesis in sun‐demanding *C. arundinacea* was found only during the spring months when the tree canopy was not fully developed, and the plants were exposed to relatively high DLI values. Finally, our results imply that understorey vegetation in the future could gain more importance in carbon sequestration and other ecosystem functions as it shows less evidence of photosynthetic downregulation, improved water use efficiency, enhanced amount of carbon accumulated in the biomass, particularly roots, and also high plasticity to changing light conditions given mainly by species‐specific differences in the dynamics of development and shade‐tolerance.

## CONFLICT OF INTEREST

None declared.

## AUTHOR CONTRIBUTIONS

P.H., K.K., and O.U. conceived the ideas, designed methodology, and analyzed data; P.H. and K.K. collected data; P.H. and O.U. led the writing of the manuscript assisted by S.L. and K.K. All authors contributed critically to the drafts and gave final approval for publication.

## Data Availability

All data used in this manuscript are presented in the figures and supporting information.
