# Supplementary figures and images for: Distinct seasonal dynamics of responses to elevated CO2 in two understorey grass species differing in shade‐tolerance
Source: Ecol Evol. 2019 Nov 29;9(24):13663–77. doi: 10.1002/ece3.5738 (PMC6953567; doi:10.1002/ece3.5738)

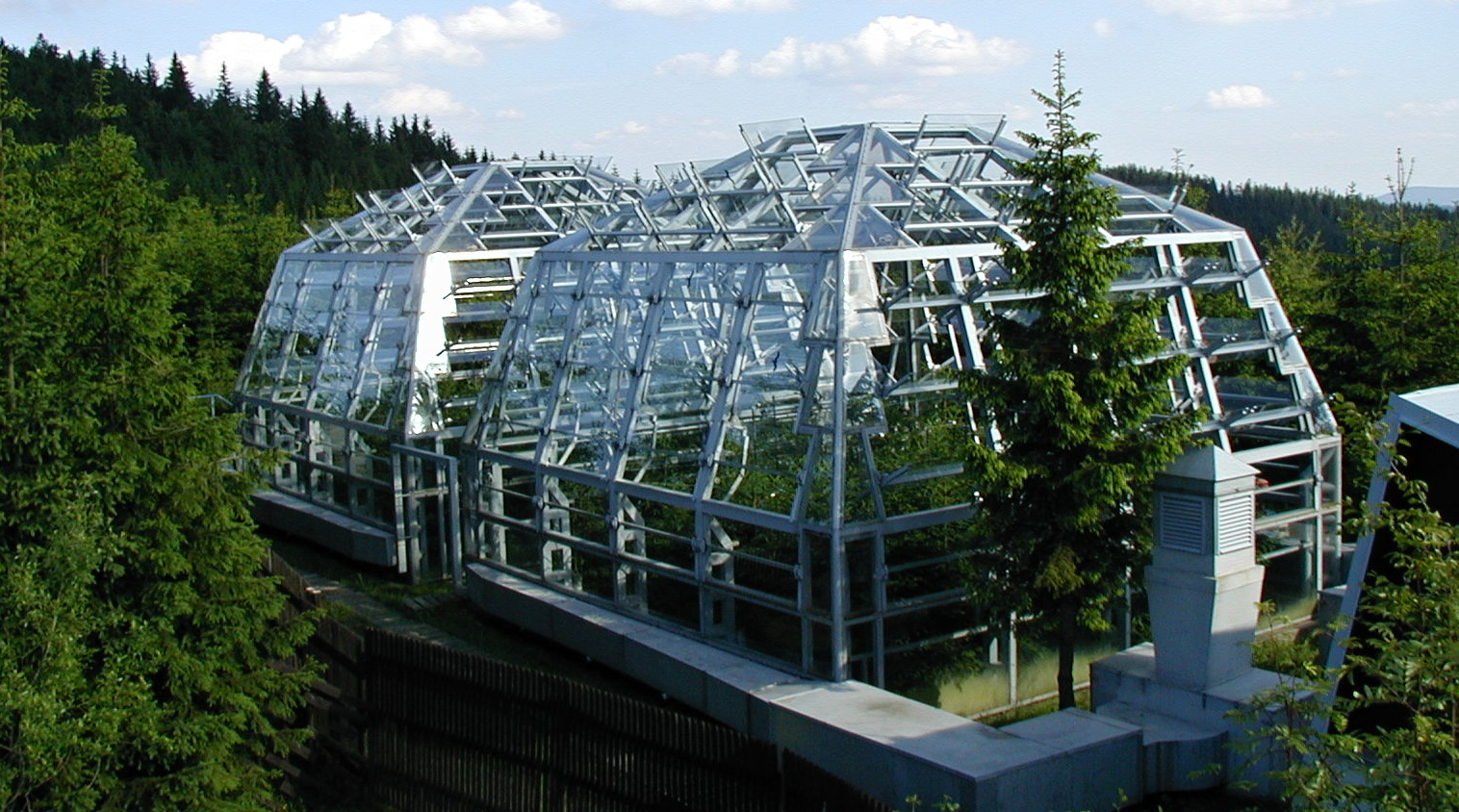

Supplement: Supplementary file 1 [file ECE3-9-13663-s001.tif]

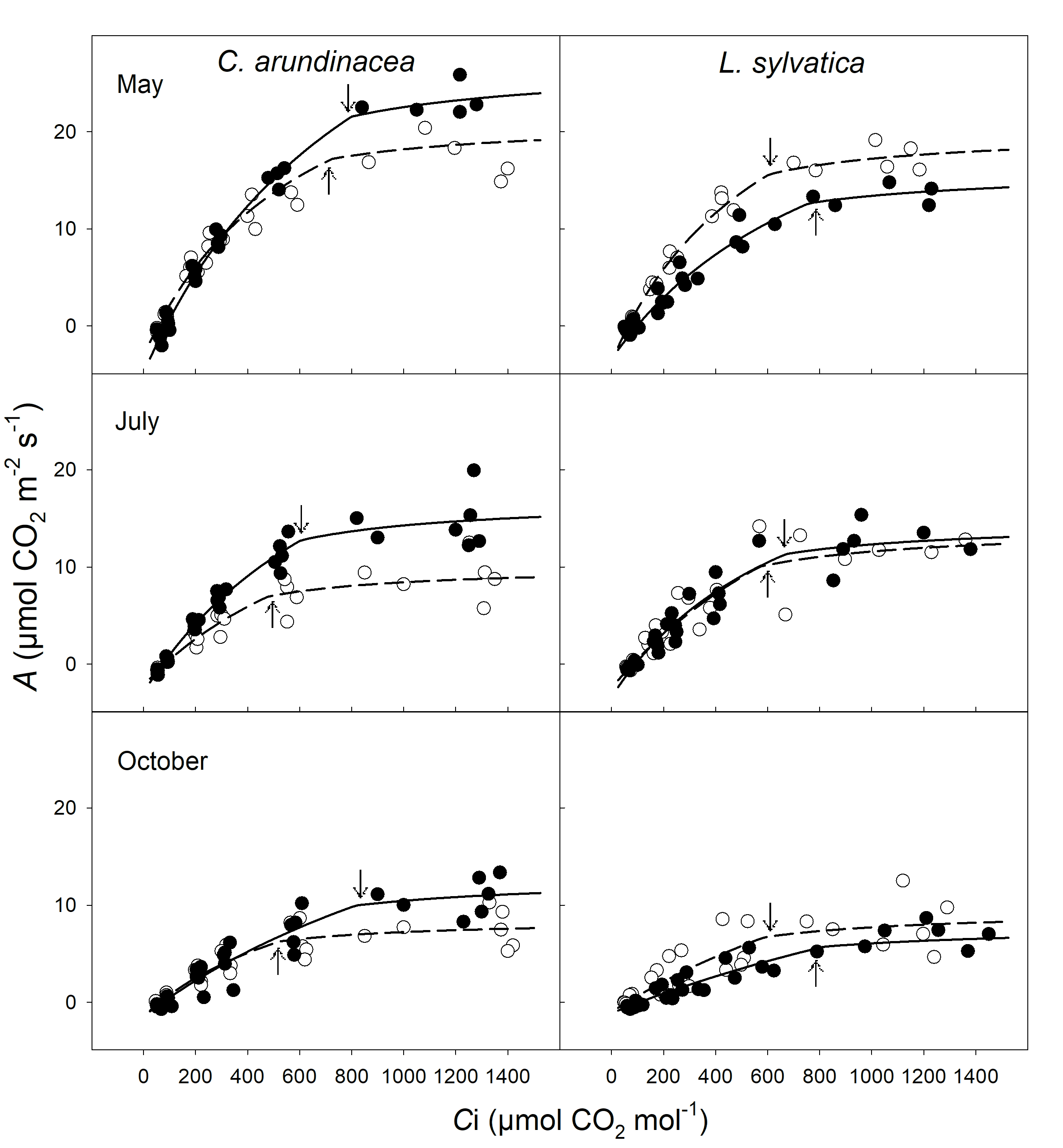

Supplement: Supplementary file 2 [file ECE3-9-13663-s002.TIF]

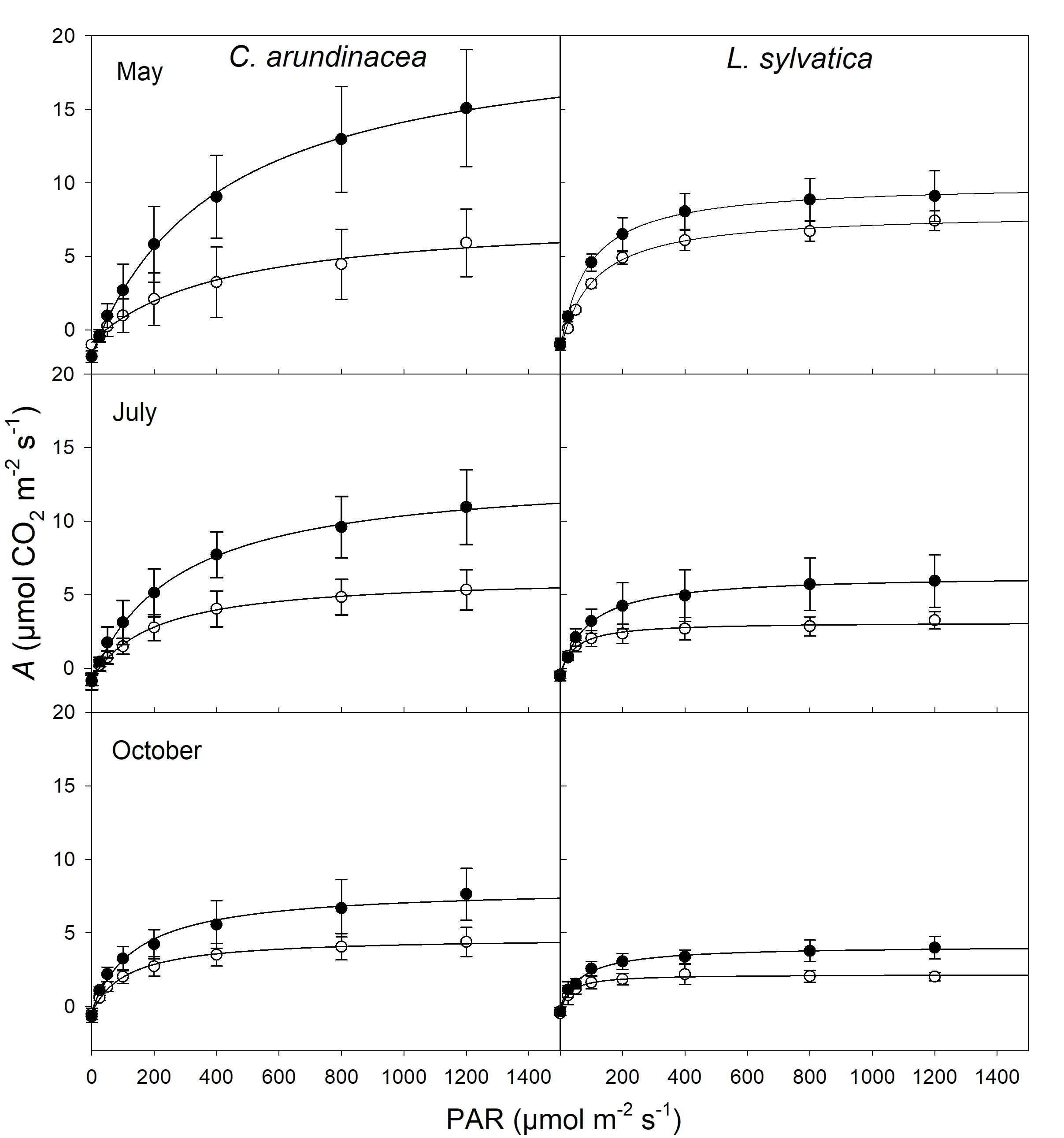

Supplement: Supplementary file 3 [file ECE3-9-13663-s003.TIF]

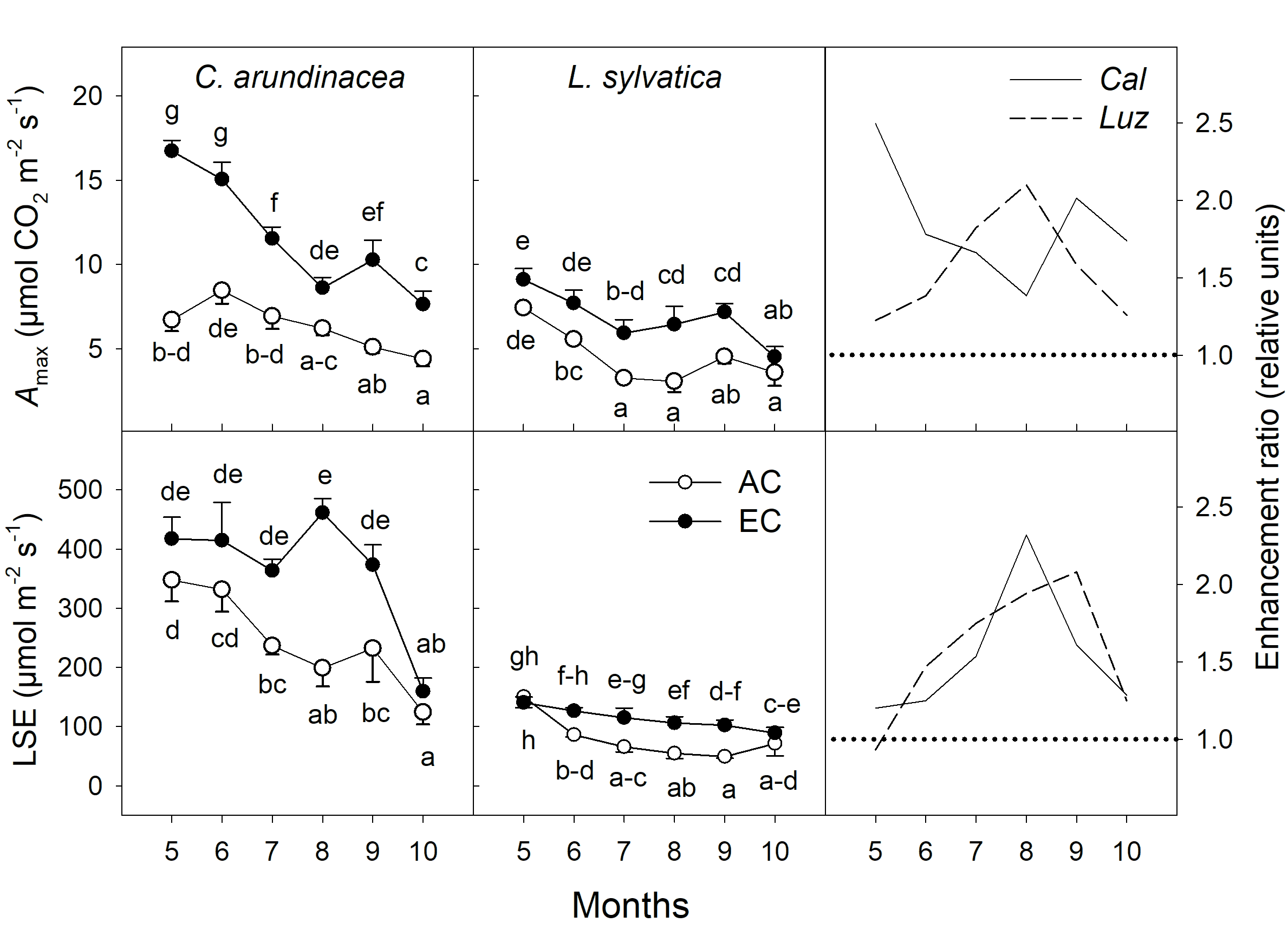

Supplement: Supplementary file 4 [file ECE3-9-13663-s004.TIF]
